# Bioautography and GC-MS based identification of piperine and trichostachine as the active quorum quenching compounds in black pepper

**DOI:** 10.1016/j.heliyon.2019.e03137

**Published:** 2020-01-02

**Authors:** Juan Vázquez-Martínez, Génesis V. Buitemea-Cantúa, Juan Manuel Gutierrez-Villagomez, Julia P. García-González, Enrique Ramírez-Chávez, Jorge Molina-Torres

**Affiliations:** a Departamento de Ingeniería Bioquímica, Instituto Tecnológico Superior de Irapuato (ITESI), Tecnológico Nacional de México (TecNM), Irapuato, Guanajuato, Mexico; b Departamento de Biotecnología y Bioquímica, Centro de Investigación y de Estudios Avanzados del IPN (CINVESTAV-IPN) Unidad Irapuato, Irapuato, Guanajuato, Mexico; c Centro de Biotecnología-FEMSA, Escuela de Ingeniería y Ciencias, Tecnológico de Monterrey, Monterrey, Nuevo León, Mexico; d Institut National de la Recherche Scientifique (INRS), Centre Eau Terre Environnement, Quebec City, Quebec, G1K 9A9, Canada

**Keywords:** Food science, *N*-Acyl homoserine lactone, *Piper nigrum*, Piperamide, Quorum quenching, Quorum sensing

## Abstract

In the search of new and safe antibacterial compounds, the quorum sensing system (QS) modulation by natural products has been studied. As a result, many plant-derived compounds have been identified as potent quorum sensing inhibitors. *Piper nigrum* L. (black pepper) ethanolic extract inhibits the QS in some Gram-negative bacteria but the active components have not been previously identified. Thus, the objective of this work was to identify the *P. nigrum* peppercorns main components that block the QS, applying bioassay and chromatographic techniques. Piperine and trichostachine were identified as the main components responsible for the quorum quenching (QQ) activity of *P. nigrum* peppercorns extract. Piperine at 30 mg/L, decreased the violacein production by *Chromobacterium violaceum* CV026 by 35%, without affecting bacterial growth. Piperine concentration of 40 mg/L decreases violacein production by *C. violaceum* CV026 by 70% and growth in only 4.34%. Trichostachine at 50 mg/L decreases violacein production by *C. violaceum* CV026 by 12%, without affecting bacterial growth. *P. nigrum e*xtract concentration of 0.5 g/L decreased violacein production in 40 % and no effects on growth were observed. Neither *P. nigrum* extract, piperine, nor trichostachine did affect QS of *Pseudomonas aeruginosa* PAO1. Data here described exhibit the potential of piperamides as modulators of QS, not previously reported.

## 1 Introduction

Microbial communication comprises the coordinate response mechanisms to changes in the environment, that some microorganisms have developed. The quorum sensing system (QS) is the most studied microbial communication mechanism (Fuqua and Greenberg, 2002; Abisado et al., 2018). Via QS, microorganisms evaluate their cell density and, above a threshold level, trigger collective development changes through synthesis, detection and response to chemical signals known as autoinducers (Nealson, 1977; Fuqua and Greenberg, 2002). One of the autoinducer-compounds family synthesized by Gram-negative bacteria is the denominated acyl homoserine lactones (AHLs) (Schuster et al., 2013). The Gram-negative soil bacterium *Chromobacterium violaceum*, in response to QS, modulates its violacein production, a purple antibiotic. The main autoinducer produced by *C. violaceum* ATCC 31532 is the *N*-hexanoyl homoserine lactone (C6-HSL) (McClean et al., 1997). The mutant strain *C. violaceum* CV026 (sometimes mistakenly called CVO26) lacks the autoinducer synthase CviI but express the autoinducer receptor, thus it requires the exogenous addition of C6-HSL to carry out QS and to produce violacein (McClean et al., 1997). Then, CV026 is useful to detect compounds that induce or inhibit the QS (Choo et al., 2006). In a similar way, the opportunistic pathogen *Pseudomonas aeruginosa* possesses two AHL dependent QS systems: the *las* and *rhl* (Chong et al., 2011). The strain *P. aeruginosa* PAO1 is useful to detect QS active compounds against biofilm and virulence factors production (Chong et al., 2011). The study of QS has become an important topic due to its relationship with pathogenic multi-resistant-antibiotic microorganisms (Defoirdt, 2018).

Some plants and microorganisms produce compounds that can interfere with the bacterial QS. This QS interference is known as quorum quenching (QQ). Some natural compounds, which modulate the cellular processes dependent on QS, have been studied in recent years due to their impact on biotechnology and medicine (Scopel et al., 2017). Plant extracts such as *Amomum tsaoko* (Rahman et al., 2017), *Allium sativum* (Harjai et al., 2010), *Phyllanthus amarus* (Priya et al., 2013), *Medicago truncatula* (Gao et al., 2003), *Vanilla planifolia* (Choo et al., 2006), *Piper betle* (Datta et al., 2016; Srinivasan et al., 2016, 2017); *Piper delineatum* (Martín-Rodríguez et al., 2015) and *Piper nigrum* (Tan et al., 2013; Dazal et al., 2015), among others, have been reported to display QQ activity. However, the identification of the active components responsible for this activity has not been accomplished. The efforts have been directed to find effective and safe QQ natural compounds (Quecan et al., 2019; Rivera et al., 2019). As a result, there are some reports describing the identification of the active QQ compounds from plant extracts, *v.gr.* malabaricone C isolated from *Myristica cinnamomea* (Chong et al., 2011), menthol from *Mentha piperita* (Husain et al., 2015) and luteolin from *Capsicum frutescens* (Rivera et al., 2019). Thus, the objective of this study is to identify the main components responsible for the QQ in the *P. nigrum* peppercorns, not previously described.

## 2 Materials and methods

### 2.1 Plant material and extract preparation

*Piper nigrum* (black pepper) peppercorns were bought at a local market in Irapuato, Guanajuato, Mexico (20°40′27.6″N 101°20′44.5″W) and 20 g were triturated in mortar. The triturated material was extracted with 100 mL of absolute ethanol (99.8%, Sigma-Aldrich, USA) in an extraction unit model E-816 (Büchi, Flawil, CHE). The extract was solvent freed in a rotary evaporator model RE-111 (Büchi, Flawil, CHE) at 45 °C and low pressure. The residue weight was 332.4 mg and was dissolved in absolute ethanol to obtain a concentration of 0.5 g/mL.

### 2.2 Microorganisms and growth conditions

*Chromobacterium violaceum* CV026 was obtained from the CINVESTAV-IPN campus Zacatenco culture collection (Mexico City, MEX). A preinoculum of 50 mL was prepared in Luria-Bertani-Miller or LB-10 broth (tryptone 10g, yeast extract 5 g, NaCl 10 g, deionized water 1L) supplemented with kanamycin to a final concentration of 25 mg/mL. For the QQ assays, *C. violaceum* CV026 was grown in LB-10 broth and C6-HSL was added to a final concentration of 25 μM. Cultures were incubated at 30 °C at 150 rpm (Choo et al., 2006; Chu et al., 2011). *Pseudomonas aeruginosa* PAO1 was kindly supplied by Dr José Lopéz-Bucio (IIQB, UMSNH, Morelia, MEX). *P. aeruginosa* PAO1 preinoculum was grown in Luria-Bertani-Lennox or LB broth (tryptone 10 g, yeast extract 5 g, NaCl 5 g, deionized water 1 L) at 37 °C and 150 rpm. For semi-solid medium preparation LB-10 broth was added with agar (Sigma-Aldrich, USA) at final concentration of 0.7%.

### 2.3 Thin-layer chromatography

Thin-layer chromatography (TLC) analysis was conducted on preparative 20 × 20 cm plates without fluorescent indicator (ANALTECH, Newark, DE, USA). An aliquot of 100 μL of the extract was applied as an 18 cm long band, two centimeters above the lower edge of the plate using an automatic sampler model ATS4 (CAMAG, Muttenz, CHE). Each plate was developed in a pre-saturated glass chamber containing a saturation paper sheet of 20 × 20 cm. The mobile phase was hexane:ethyl acetate (2:1), the same mobile phase composition was used in all the posterior TLC assays. In order to obtain a better resolution, plates were developed twice using the same solvent system: for the first development the solvent front reached 10 cm and for the second development the solvent front reached 18 cm. The digital image acquisition of the developed plates was done with an automatic TLC visualizer (CAMAG, Muttenz, CHE). TLC data was processed using the WinCATS Planar Chromatography Manager software (CAMAG, Muttenz, CHE). Application and development were done in quadruplicate, two plates were used for bioautography assays and two plates were used for the active component detection. Developed plates were evaluated under 366 nm UV light and under white light after the bioautography.

### 2.4 Bioautography assays

For bioautography assays, an aliquot of 50 mL of *C. violaceum* CV026 preinoculum was mixed with 100 mL of semi-solid LB-10 agar to a final optical density at 600 nm (OD_600nm_) of 0.1, containing C6-HSL at 25 μM and kanamycin at 8.3 mg/L. The presence of kanamycin on TLC was maintained in order to avoid the growth of contaminant microorganisms since the TLC plates were not sterilized. This medium mix was spread on the developed and solvent-freed plates. The semi-solid LB-10 agar layer was about 3 mm thick. Plates were incubated at 27 °C in a humid chamber for 24 h (Vazquez-Martinez et al., 2018; Chu et al., 2011). Once the *C. violaceum* CV026 violacein production on the plate was documented, 10 mL of a 5 mM 2, 3, 5-triphenyltetrazolium chloride (TTC) solution was sprayed on the agar surface and the plate was incubated for 1 h at 27 °C in a humid chamber. This was done in order to differentiate the effect between growth and QS since the TTC produces the red-coloured compound 1,3,5 triphenylformazan when it is enzymatically reduced by metabolically active cells indicating cell viability (Seidler and van Noorden, 1994).

### 2.5 Active components detection

For active components detection, the solvent freed developed TLC plates were observed under 366 UV light and were compared with the bioautography-assay plates. The bands that corresponded to the inhibition zones were scraped from the plates. Components were eluted from the silica with 1.5 mL of absolute ethanol. Each eluted band-compounds were ethanol freed to dryness under nitrogen gas stream and dissolved in 300 μL of absolute ethanol. Then, 150 μL of each fraction was purified on silica-gel aluminium sheets with fluorescein as fluorescent indicator (Fluka Analytical, Sigma-Aldrich Co., DE). After, purified bands were scraped, eluted, dried, dissolved in 100 μL of absolute ethanol, and stored at -20 °C until further analysis by gas chromatography coupled to a quadrupole triple axis detector with electronic impact ionization (GC-EIMS).

### 2.6 GC-EIMS analysis and compound identification

A gas chromatograph (GC) model 7890A coupled to a quadrupole triple-axis detector with electronic impact ionization (EIMS) model 5975 (Agilent Technologies, Inc., USA) equipped with a capillary column DB-1MSUI (60 m × 250 μm × 0.25 μm, J&W, Agilent Technologies, Inc., USA) was employed for compounds analysis. An aliquot of 1 μL of the sample was injected in splitless mode. Injection temperature was 250 °C. Helium was used as carrier gas with a constant flow of 1 mL/min. The GC oven program started at 150 °C and held for 3 min, then increased at 4 °C/min to 300 °C and held for 20 min. The transfer line temperature was 250 °C. Temperature of the ion source and the quadrupole was 230 °C and 150 °C, respectively. Measurements were performed in SCAN mode with a mass range of 50–550 and operated at 2.9 scans per second. Mass spectra were obtained at 70 eV. Data was collected with the MassHunter Workstation version B.06.00 software (Agilent Technologies, Inc., USA). Retention time and mass spectrum of each component were determined with the Automated Mass Spectral Deconvolution and Identification System "AMDIS" software (http://www.amdis.net/). Compounds were identified using the mass spectra database and library NIST MS Search software version 2.0 (National Institute of Standards and Technology, USA) and/or the respective standard, for piperine standard purity = 97% and *m/z*-relative abundance % = (115-100%, 201-91%, 285-71%, 173-36%, 202, 26%, 174-25%, 171-24%, 116-22%), see Supplementary material.

### 2.7 Trichostachine purification

To obtain enough of high-purity trichostachine -one of the identified active components-for the QQ assays, this compound was isolated and purified from the *P. nigrum* extract. The purification was performed as follows: 4 g of dried *P. nigrum* extract was mixed with 2 g of silica gel 20–200 mesh (Sigma-Aldrich Co., DE, USA). An open column was prepared by loading a 2 cm × 45 cm glass column with silica gel 20–200 mesh suspended in cold hexane so that the column packing was approximately 35 cm long. The column was flushed with 20 mL of cold hexane, and then loaded with the silica/extract mix and the sample was allowed to pass through the column by gravity. Once the sample was loaded, cold hexane was added until 50 mL was recovered from the column. Then, a gradient of hexane:ethyl acetate (80:20, 60:40, 50:50, 40:60 20:80), 100 mL of each solution was passed through the column. Finally, 200 mL of ethyl acetate was added to the column. Trichostachine was monitored through the chromatographic column as a small blue-green band, just after the big green band of piperine, illuminating with 360 nm light using a Cole-Parmer UV lamp. Under visible light, trichostachine appears as a small pale-yellow band after the big yellow band of piperine. Once the trichostachine was eluted from the column, the fraction was solvent freed in a rotary evaporator and dissolved in 1 mL of absolute ethanol. After that, all this solution was used to purify the trichostachine on aluminum TLC plates. Then, the purified band of trichostachine was scraped, eluted, dried, quantified and stored at −20 °C until activity analysis. Purity was monitored via GC-EIMS using the conditions described in section 2.6.

### 2.8 Quorum quenching activity of the *P. nigrum* extract

#### 2.8.1 Effect on violacein production by *C. violaceum* CV026

This assay was performed in a polypropylene, round-bottom, 96-well, deep-well microplate (Axygen, Corning Inc., USA). One mL of *C. violaceum* CV026 inoculum at final OD_600nm_ of 0.1 was mixed with an aliquot of *P. nigrum* crude extract in each well. A concentration series of 0.5, 1, 1.5, 2 and 2.5 g of extract/L of culture medium was tested. In order to increase the solubility of active components, dimethyl sulfoxide (DMSO) was added as recommended by Cao et al. (2009). DMSO and ethanol concentration was adjusted to 0.5% v/v in all treatments. In addition, the growth and/or violacein production were also evaluated in the presence and absence of DMSO at 0.5% v/v, ethanol at 0.5% v/v and C6-HSL (25 μM) without the addition of the extract. The 96-well plate was incubated under orbital agitation (Thomas Scientific, USA) of 300 rpm for 48 h at 30 °C. After this incubation time, a 100 μL culture aliquot of each treatment was used to determine growth and violacein production using clear polystyrene 96-well plates. To estimate growth, the OD_600nm_ of each sample was measured using a microplate reader spectrophotometer (Bio-Rad Inc, USA). Violacein was extracted with absolute ethanol and its concentration in each sample was measured at 585 nm in a microplate reader spectrophotometer (Bio-Rad Inc, USA). Five replicates of each treatment were evaluated. This method was miniaturized from the methods described by Cao et al. (2009) and Rettori and Durán (1998). Miniaturization and microscale experiments represent efficient alternatives for low-cost and less residue production (Algburi et al., 2017), consequently less environmental impact research.

#### 2.8.2 Effect on biofilm formation by *P. aeruginosa* PAO1

This assay was conducted using the method reported by Chong et al. (2011), with some modifications: an overnight culture of *P. aeruginosa* PAO1 was diluted with LB broth to obtain an OD_600nm_ of 0.1. Then, aliquots of 2 mL were mixed with an aliquot of *P. nigrum* crude extract in 15 mL sterile test tubes. A concentration series of 0.5, 1, 1.5, 2 and 2.5 g of extract/L of culture medium was tested. DMSO and ethanol concentration was adjusted to 0.5% v/v in all treatments. In addition, the growth and biofilm production were also evaluated in the presence and absence of DMSO at 0.5% v/v, and ethanol at 0.5% v/v without addition of the extract. Tubes were incubated without agitation for 24 h at 37 °C. After this, planktonic bacteria were discarded, and the biofilms were gently washed with sterile water. After discarding the wash water, biofilms were air-dried for 15 min and stained with 0.5 mL of a 1% (v/v) crystal violet solution for 45 min. The stained biofilms were gently washed with sterile water. After removing the wash water, 1 mL of 95% ethanol was added in order to extract the crystal violet absorbed in the biofilm matrix. For measuring, 100 μL of the resulting stained solutions were transferred to a 96-well microplate and its absorbance at 590 nm was determined in a microplate reader spectrophotometer (Bio-Rad Inc, USA).

#### 2.8.3 Effect on pyocyanin production by *P. aeruginosa* PAO1

This assay was conducted using the method reported by Essar et al. (1990). *P. aeruginosa* PAO1 was cultivated under the same conditions described in section 2.8.2 for the quantification of biofilm formation by *P. aeruginosa* PAO1, with the only difference that the tubes were incubated under orbital agitation of 250 rpm for 24 h at 37 °C. Briefly, an aliquot of 500 μL of the cells-free broth of *P. aeruginosa* PAO1 culture was extracted with 300 μL of chloroform and then, the chloroform was extracted with 100 μL of 0.2 M hydrochloric acid (HCl). Pyocyanin content was measured at 520 nm in a microplate reader spectrophotometer (Bio-Rad Inc, USA). Pyocyanin, in acidic solution, gives a pink to deep-red hue.

### 2.9 Quorum quenching activity of piperine and trichostachine

Ethanolic solutions of each, piperine standard and purified trichostachine, were prepared at a concentration of 10 mg/mL. The effect of each compound over QS in *C. violaceum* CV026 and *P. aeruginosa* PAO1was tested at concentration series from 10 to 50 mg/L culture medium, and the same methodologies described in section 2.8 were applied.

### 2.10 Molecular docking of piperine and trichostachine with CviR receptor

Molecular docking analysis was performed according to Sun et al. (2016), Ravichandran et al. (2018) and Rivera et al. (2019), with some modifications. Crystalized structure of the receptor CviR of *C. violaceum* ATCC 12472 bonded to the ligand C6-HSL (PDB ID: 3QP6; Chen et al., 2011) was obtained from the RCSB-Protein Data Bank database (http://www.rcsb.org/pdb/home/home.do). Molecular docking was performed between the CviR structure and, C6-HSL, piperine and trichostachine, as ligands. Docking calculations were carried out using the Schrodinger software (Maestro v12.1, Glide tool). Protein and ligands were pre-processed using the Protein Preparation Wizard and the Virtual Screening Workflow tools, including Ligand Preparation and Grid Generation tools. Docking analysis was performed using the Grid Based Ligand Docking with Energetics (GLIDE) module. Grid boxes were fixed by “picking up” the native ligand C6-HSL and adjusted for ligands with the same size of C6-HSL and for ligands bigger than this. After analysis, ribbons, surface and interacting-residues docking representations were generated and fixed to obtain the best view in each case. Docking scores and hydrogen bonds number were determined and used to establish the best docking ligand.

### 2.11 Statistical analysis

Data are presented as mean ± SD. The Shapiro-Wilk normality and Levene's equal variances tests were performed. Growth data analysis was conducted comparing treatments through a one-way analysis of variance (ANOVA) followed by a post-hoc Tukey test. Violacein, biofilm and pyocyanin production data were analysed using an ANOVA-Welch followed by a post-hoc Dunnett T3 test for normally distributed data with unequal variances. The α-value was set at less than 0.05. Statistical analysis was performed using the SPSS Statistics software version 22 (IBM, USA).

## 3 Results and discussion

### 3.1 Active compounds identification

**Figure 1:**
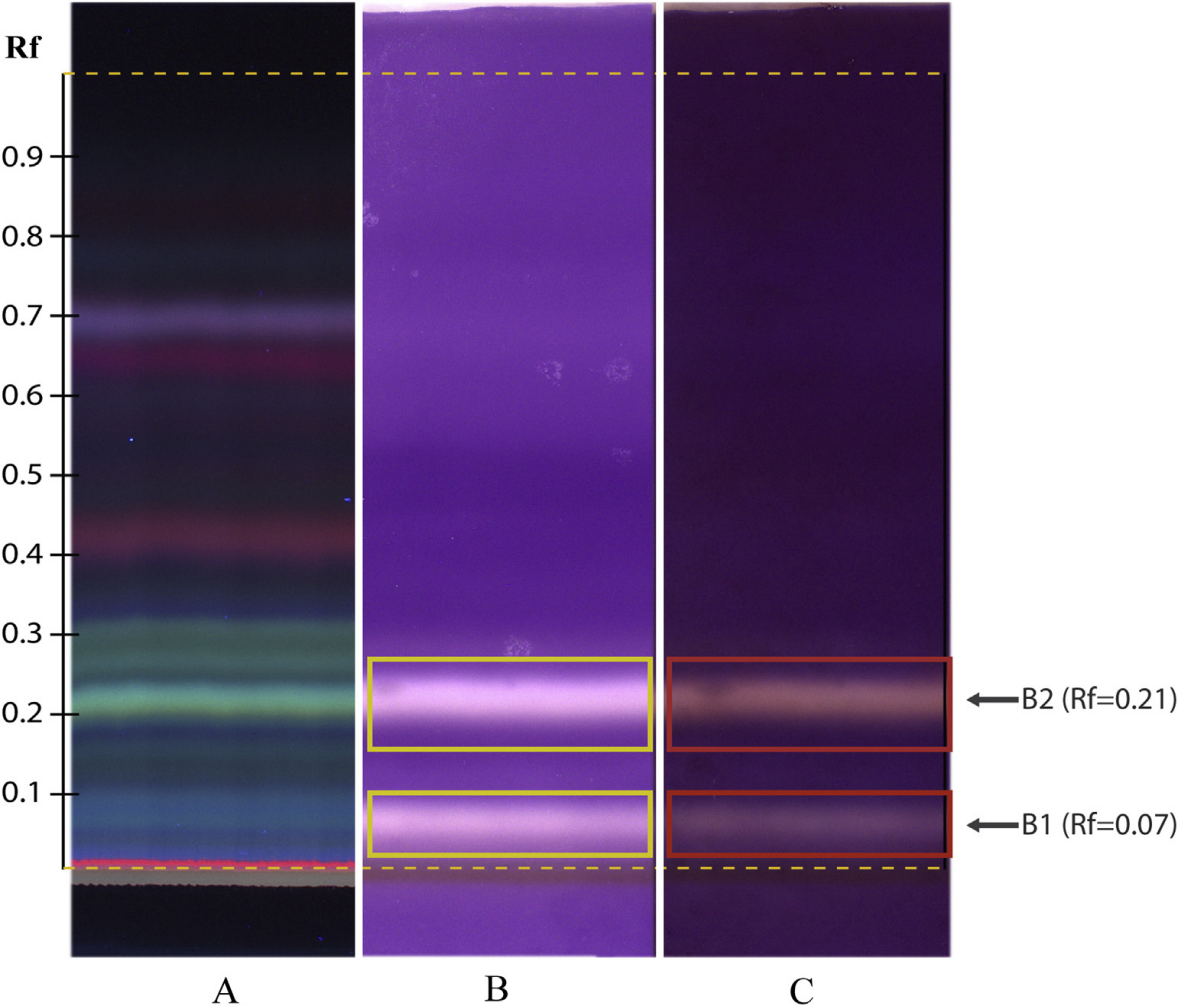
TLC chromatograms of A) *P. nigrum* ethanolic extract observed under 366 nm UV light; B) Inhibition of violacein production in *C. violaceum* CV026 by *P. nigrum* extract (yellow rectangles); C) TTC reduction by *C. violaceum* CV026 active cells (red rectangles). Quorum quenching active components were retained in two bands, B1 and B2, at Rf = 0.07 and 0.21 respectively.

The thin layer chromatography (TLC) analysis of the *P. nigrum* extract, showed two blue-green fluorescent bands under 366 UV illumination at retention factor (Rf) of 0.07 and 0.21 (Figure 1A). After *C. violaceum* CV026 bioautography assay, these bands displayed a white colouration (Figure 1B) indicating violacein synthesis inhibition and were considered as candidates for QQ active components localization. The blue-green fluorescence of the band at Rf = 0.21 is consistent with the blue fluorescence of piperine, the characteristic compound of *P. nigrum*, previously described (Ikan, 1991). Subsequent to the 2, 3, 5-triphenyltetrazolium chloride (TTC) application, these two bands acquired a pink hue (Figure 1C). This colouration change is due to TTC reduction by the active cells of *C. violaceum* CV026, as interpreted by Seidler and van Noorden (1994). These results indicate that the components retained at Rf of 0.07 and 0.21 are the main compounds responsible for the QQ in the *P. nigrum* extract, since these compounds inhibited the violacein production but not the bacterial growth. In the same assay, other bands with higher Rf displayed lower QQ and were not considered for the following experiments.

**Figure 2:**
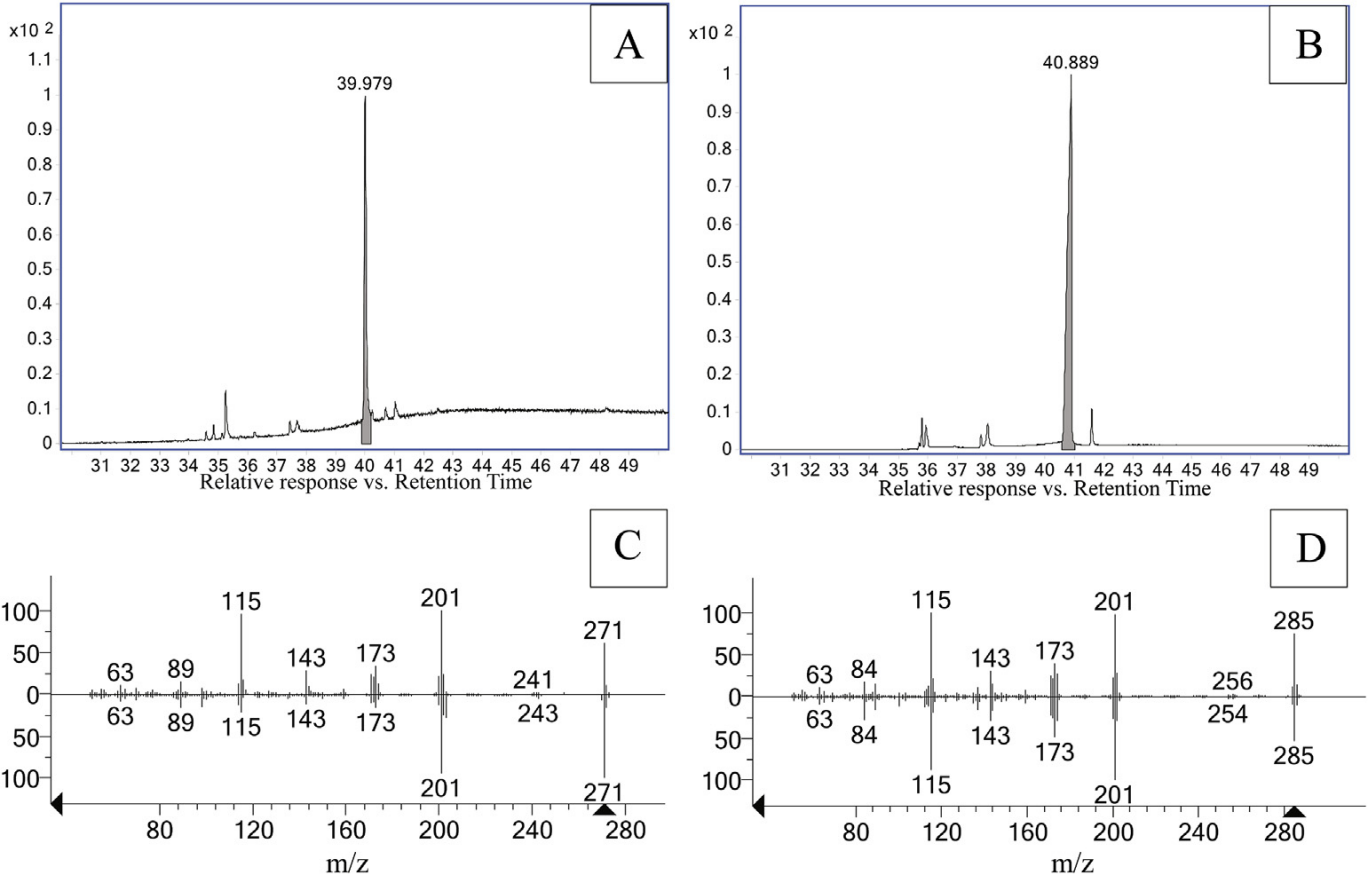
Total ion chromatograms of purified components eluted from TLC-bands retained at Rf = 0.07 (A) and Rf = 0.21 (B). The respective main peaks correspond to trichostachine (Rt = 39.979) (A) and piperine (Rt = 40.889) (B), respectively. Compound identification was done through the comparison of extracted mass spectra (upper) and the NIST-library mass spectra (lower), for trichostachine (C) and piperine (D).

The components of each of the two QQ candidate bands were eluted, purified and later analysed by GC-EIMS. Each one of them displayed a single main peak in their corresponding chromatograms (Figure 2A and B). The elute of the band with Rf of 0.07 showed a peak at retention time of 39.979 min (*m/z*-relative abundance = 201-100%, 115-93%, 271-61%, 173-33%, 202-23%, 171-23%, 200-21%, 172-21%) and the elute of the band with Rf = 0.21 showed a peak at 40.889 min (*m/z*-relative abundance = 115-100%, 201-97%, 285-75%, 173-39%, 202-28%, 174-27%, 171-24%, 200-23%). The mass spectrum of each peak was processed with AMDIS software and produced an m/z ions purity of 81 and 91, respectively. The extracted mass spectra at 39.979 and 40.889 were compared with the mass spectra of the NIST software and database. The best matches were trichostachine and piperine with values of 817 and 927, respectively (Figure 2C and D). Thus, it was concluded that these are the main compounds responsible for the inhibition of violacein production by *C. violaceum* CV026. There are not previous reports of these compounds or other piperamides as QS inhibitors.

**Figure 3:**
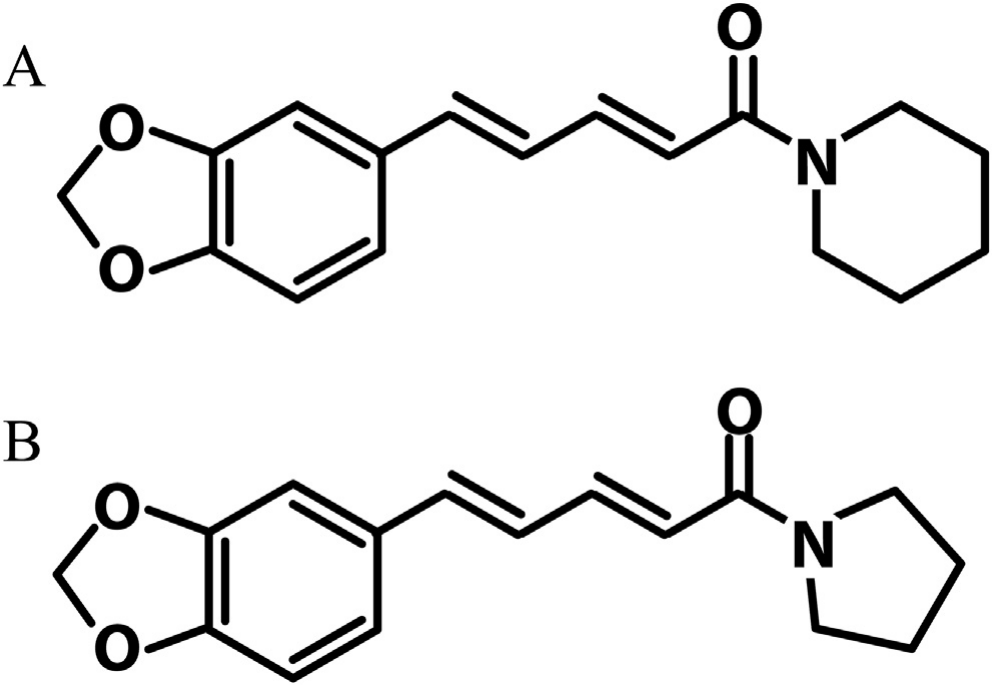
Chemical structures of piperine (A) and trichostachine (B).

Piperine and trichostachine chemical structures are closely related. The structural difference is that the piperidine ring in piperine is substituted by a pyrrolidine ring in trichostachine (Figure 3). The 6-membered ring of piperidine (including nitrogen) is derived from lysine, and the 5-membered ring of pyrrolidine (including nitrogen) derives from ornithine (Seigler, 1998). Piperine and trichostachine share some biosynthetic characteristics: both result from the condensation of piperoyl-CoA with a cyclic amine, linked by an amide bond (Geisler and Gross, 1990). Therefore, the QQ activity of these compounds could be related to their common characteristics, the amide bond and/or the piperic acid substituent. Thus, it is probable that other piperamides or similar chemical structures also can modulate QS.

Piperine and trichostachine are alfa-unsaturated acylated amides (Boonen et al., 2012) and, since they contain a nitrogen heterocycle in their structure, are also classified as alkaloids. Along with other acylated amides and alkaloids, piperine and trichostachine have a wide range of biological activities *e.g.* antifungal and antibacterial (Seigler, 1998; Srinivasan, 2007). Other alkamides such as afinin and *N*-isobutyl-2E-decenamide have antifungal and antibacterial activity (Molina-Torres et al., 2004). It has been observed that piperine inhibits biofilm production in the Gram-positive bacterium *Streptococcus mutans* (Dwivedi and Singh, 2016). Further, it is known that piperine is the compound responsible for the pungency and flavour of the *P. nigrum* peppercorns. Thus, it is interesting that these broad-spectrum bioactive compounds also modulate the Gram-negative bacterium QS.

The QQ of piperine and trichostachine could be due to their structural similarity to the AHL. It has been reported that some synthetic acylated amides structurally similar to AHLs can modulate QS (Vazquez-Martinez et al., 2018). A similar case occurs with the halogenated furanones of *Delisea pulchra*, such compounds interfere with the QS signal receptors mimicking the AHLs (Manefield et al., 1999; Rasmussen et al., 2000).

### 3.2 Quorum quenching activity measurement of *P. nigrum* extract, piperine and trichostachine

**Figure 4:**
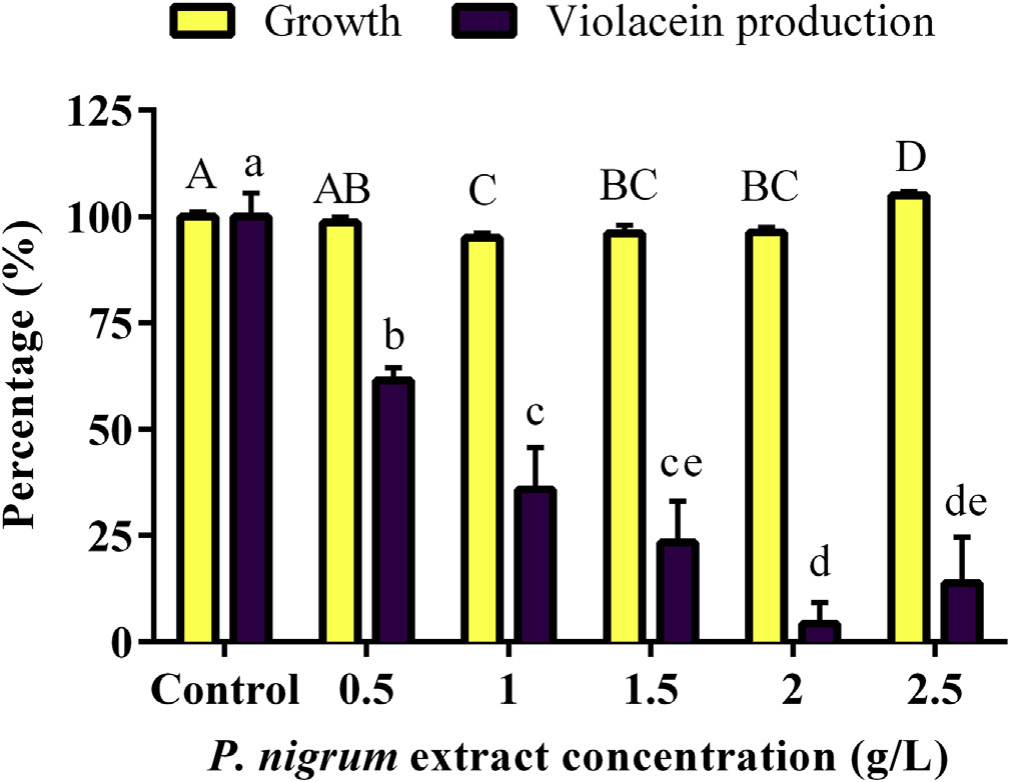
Effect of *P. nigrum* extract on growth and violacein production by *C. violaceum* CV026. Data are shown as growth (yellow bars) and violacein-production (violet bars) percentages normalized to the respective DMSO-ethanol control. Bars correspond to the mean values of five replicates and the error bars correspond to the standard deviation of the mean. Different capital letters indicate significant differences (*p* < 0.05) for growth data, and different lowercase letters indicate significant differences (*p* < 0.05) for violacein production data. Means with at least one common letter are not significantly different.

The *P. nigrum* extract, at a concentration of 1–2.5 g/L decreases slightly the *C. violaceum* CV026 growth, as shown by the OD_600nm_, being 5–10% different to the control. In contrast, the violacein production was drastically reduced, *i.e.* at 2 g/L violacein production was decreased by approximately 95% compared to controls (Figure 4, see Supplementary Data for absorbance data). The minimum inhibitory concentration of extract to reduce violacein production by 50% (MIC50) was 0.5 g/L.

**Figure 5:**
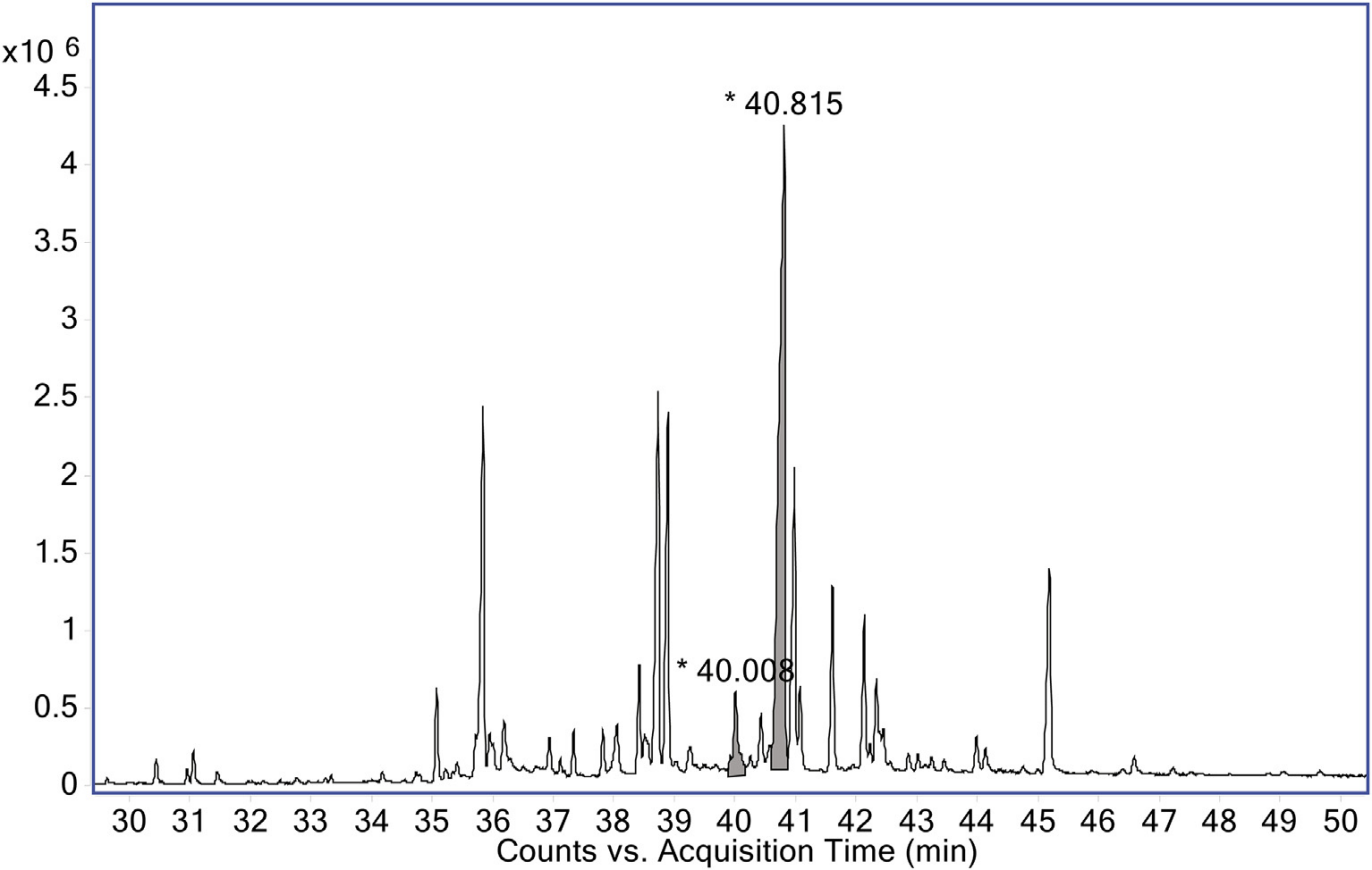
Total ion chromatogram of *P. nigrum* crude extract. Trichostachine and piperine eluting of GC at Rt = 40.008 and 40.815, respectively. Compound identification was done through the analysis of the extracted mass spectra using the software and database NIST-library mass spectra.

**Figure 6:**
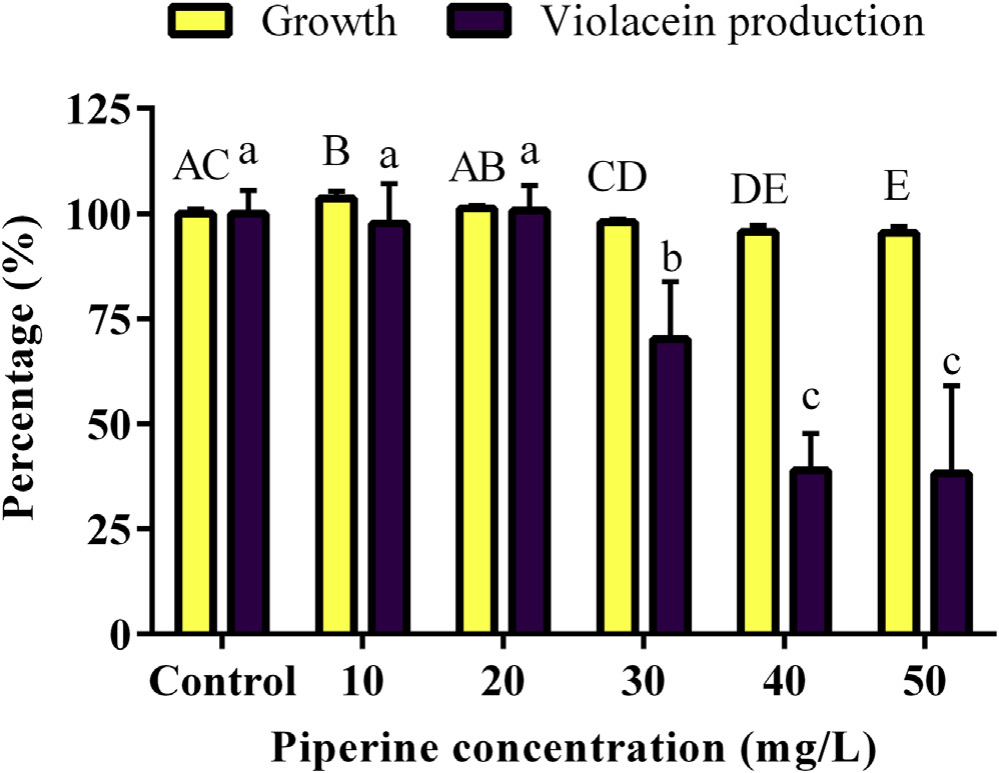
Effect of piperine on growth and violacein production by *C. violaceum* CV026. Data are shown as growth (yellow bars) and violacein-production (violacein bars) percentages normalized to the respective control with presence of 0.5 v/v DMSO and ethanol. Bars correspond to the mean values of five replicates and the error bars correspond to the standard deviation of the mean. Different capital letters indicate significant differences (*p* < 0.05) for growth data, and different lowercase letters indicate significant differences (*p* < 0.05) for violacein production data. Means with at least one common letter are not significantly different.

**Figure 7:**
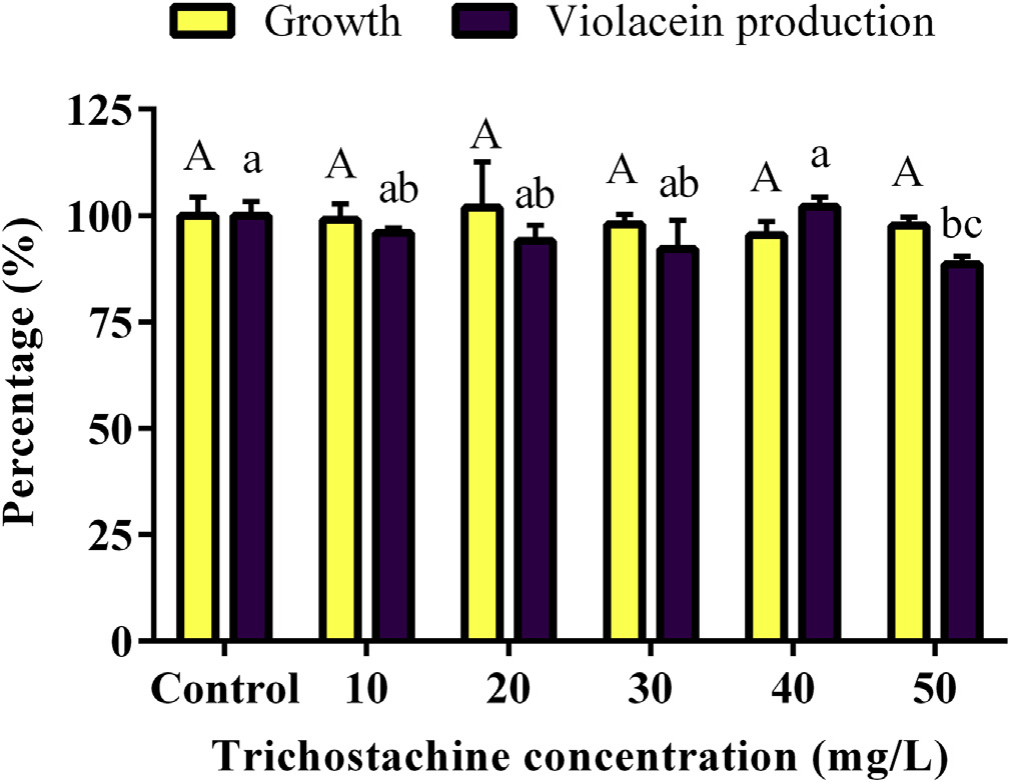
Effect of trichostachine on growth and violacein production by *C. violaceum* CV026. Data are shown as growth (yellow bars) and violacein-production (violet bars) percentages normalized to the control with presence of 0.5 v/v DMSO and ethanol. Bars correspond to the mean values of five replicates and the error bars correspond to the standard deviation of the mean. Different capital letters indicate significant differences (*p* < 0.05) for growth data, and different lowercase letters indicate significant differences (*p* < 0.05) for violacein production data. Means with at least one common letter are not significantly different.

The *P. nigrum* extract solution MIC50 corresponds approximately to 87 mg/L of piperine and 8 mg/L of trichostachine, according to their abundance in the extract determined by GC-EIMS using a calibration curve of piperine standard. In the crude extract, the piperine concentration 174 mg/g is approximately ten times lower than trichostachine concentration 16.8 mg/g. The signal response of piperine and trichostachine in the chromatogram of the *P. nigrum* extract is shown in Figure 5. The individual activity of piperine standard and purified trichostachine was tested to corroborate whether these compounds are responsible for QS inhibition. It was observed that piperine, at 40 and 50 mg/L, reduced the *C. violaceum* CV026 growth less than 5%, while at the same concentrations the violacein production was reduced approximately 70% (Figure 6, see Supplementary Data for absorbance data). Trichostachine, at 50 mg/L, does not significantly affect *C. violaceum* CV026 growth and violacein production decreases only by 12% (Figure 7, see Supplementary Data for absorbance data), being is less QQ active than piperine at the same concentration. These results prove that piperine and trichostachine affect the violacein production but not the bacterial growth in *C. violaceum i.e.* blocks the QS, something not previously described. Some concentrations of *P. nigrum* extract and piperine inhibit or induce growth, although in all cases the effect was less than 5% compared with controls (Figures 4 and 6), the concentrations that do not interfere with bacterial growth should be consider as the effective QQ concentrations (Rivera et al., 2019) *i.e*. 0.5 g/L, 30 mg/L and 50 mg/L for *P. nigrum* extract, piperine and trichostachine, respectively (Figures 4, 6 and 7). Nevertheless, concentration increment of *P. nigrum* extract and piperine affects more the violacein production than bacterial growth (Figures 4 and 6). It seems that the effect of both compounds is additive or synergistic since the inhibition percentage of the extract was not reached by any of the pure compounds, so future research is needed to test the synergistic activity of these and other compounds.

For the assay of *P. nigrum* extract, piperine and trichostachine QQ activity against *P. aeruginosa* PAO1 it was obtained a negative result: these compounds, at the tested concentrations did not affect growth, biofilm and pyocyanin production (Supplementary data). A similar negative result for pyocyanin production by *P. aeruginosa* PAO1 using only *P. nigrum* extract was previously reported (Tan et al., 2013). These data show that *P. nigrum* extract and its here identified main QQ active compounds did not affect the QS system of *P. aeruginosa* PAO1. This important difference could be due to the variation of the QS signals for each system: the signal of *C. violaceum* CV026 have a non-substituted 6-carbons acyl chain (McClean et al., 1997) whereas the signals of *P. aeruginosa* PAO1 have a non-substituted 4-carbons and beta-oxo substituted 12-carbons acyl chains (Harjai et al., 2010). Even though piperine and trichostachine do not affect the QS system in *P. aeruginosa* PAO1, the significance of the results here described resides in the observation that piperamides blocks the QS system dependent on non-substituted AHLs, something not previously reported. Besides, *C. violaceum* is a bacterium that can infect humans causing skin lesions, sepsis, and liver abscesses that may be fatal (Chattopadhyay et al., 2002). The *C. violaceum* virulence is related to its QS system (Batista and da Silva Neto, 2017; Swem et al., 2009), thus, piperamides could be used in future to combat *C. violaceum* infections. Moreover, similar QS systems to that of *C. violaceum* system are also present in *Erwinia* (Nasser et al., 1998), *Serratia* (Slater et al., 2003), and another *Pseudomonas* species (Laue et al., 2000; Miller and Bassler, 2001). The specificity of these piperamides represents an interesting trait for future work. Thus, further research is needed to test these and other piperamides in other QS systems.

### 3.3 Molecular docking analysis

**Figure 8:**
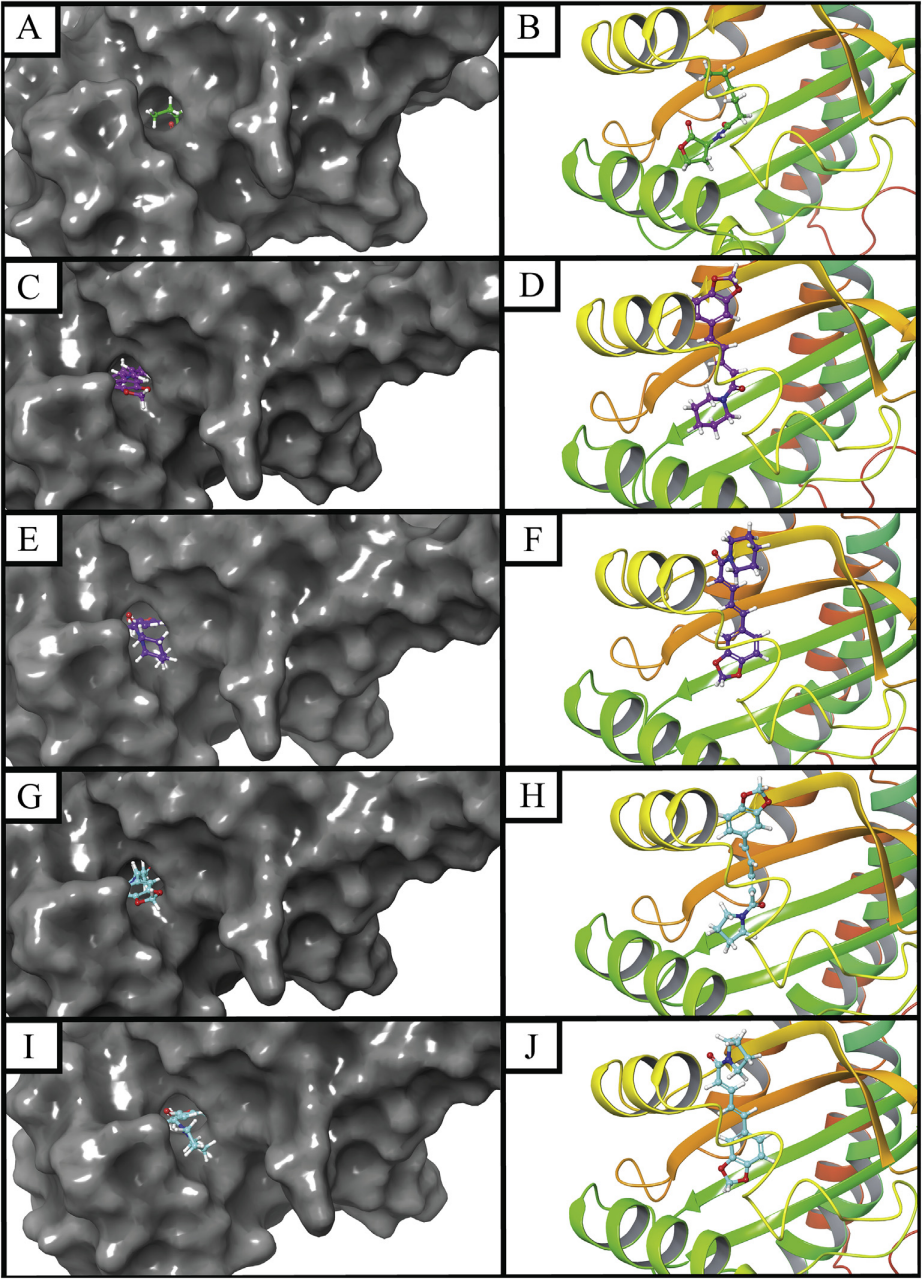
Molecular docking of CviR crystalized structure of *C. violaceum* ATCC 12472 with C6-HSL (A–B), piperine (C–F) or trichostachine (G–J). Surface (A, C, E, G, I) and backbone (B, D, F, H, J) representations of CviR for interaction with the respective ligand are showed. Small grid box size conditions were used for A-D, G and H. Big grid box conditions were used for I and J. C6-HSL green backbone, piperine purple backbone and piperine sky-blue backbone. Red atoms = oxygen. Withe atoms = hydrogen. Navy-blue atoms = nitrogen.

**Figure 9:**
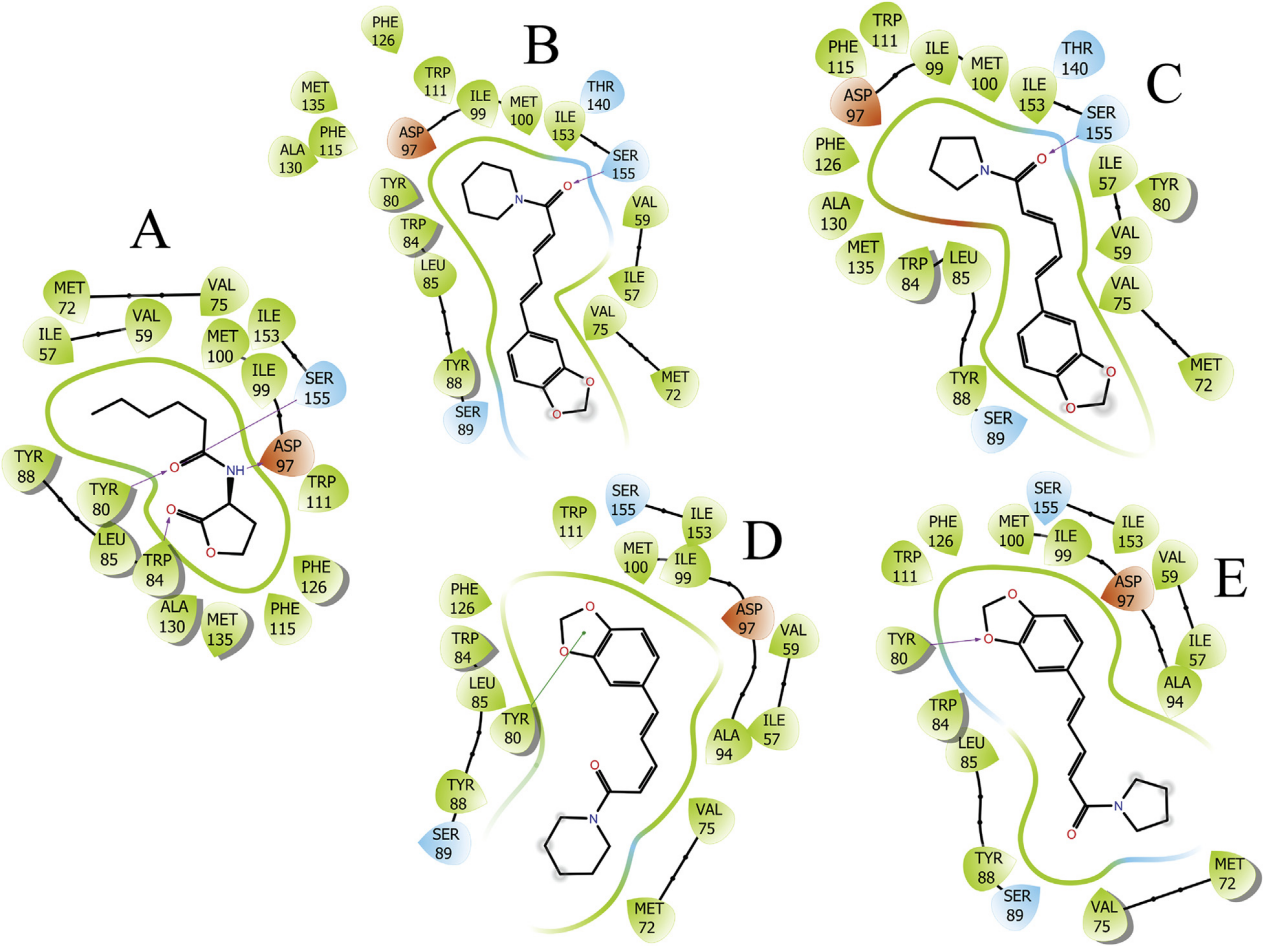
2D interaction diagrams of C6-HSL (A), piperine (B, D) and trichostachine (C, E) with CviR. Docking results for small grid box conditions are shown in B and C, and for big grid box conditions are shown in D and E. Purple arrows indicate hydrogen bonds whereas the green lines indicate Pi-Pi stacking. Sky-blue colour indicates polar interactions, green colour indicates hydrophobic interactions and orange colour indicates charge interactions. It can be noticed that all ligands interact with the Asp-97, Ser-155, Tyr-80, Trp-84, Tyr-88, Phe-115, Phe-126, Val-75, Met-135 and Leu-85 residues.

The possibilities of binding interactions between the CviR crystalized structure (PDB ID: 3QP6) from *C. violaceum* ATCC 12472 with piperine and trichostachine were assessed through *in silico* docking analysis. Molecular docking results indicate that piperine and trichostachine bind at similar level that the natural ligand C6-HSL. The docking score for the natural ligand was -8.016, this value is near to docking studies previously reported (Ravichandran et al., 2018). Docking scores using grid generation parameters to allow binding of ligands with size similar to C6-HSL (small grid box, Figure 8C, D, G, H) were -7.828 and -8.426 for piperine and trichostachine, respectively. For grid generation parameters to allow binding of ligands bigger than C6-HSL (big grid box Figure 8E, F, I, J), docking scores were -7.536 and -7.682 for piperine and trichostachine, respectively. Comparable docking scores for binding between CviR and potential QS inhibitors have been described previously (Ravichandran et al., 2018). According the molecular docking results, trichostachine should interfere better the QS system of *C. violaceum* CV026 than piperine, but the *in vitro* results showed otherwise. Despite this apparent contradiction, docking results indicates that these two piperamides accommodates in similar way inside the CviR active site in similar way (Figures 8 and 9).

The natural ligand of CviR, C6-HSL, binds forming hydrogen bonds with Tyr-80, Trp-84, Asp-97 and Ser-155, polar interactions Ser-155, and hydrophobic interactions with Ile-57, Val-59, Met-100, Ile-99, Trp-111, Phe-126, Phe-115, Met-135, Ala-130, Trp-84, Leu-85, Tyr-88, Tyr-80, Met-72, Val-75 and Ile-153 (Figure 9A). For small grid box conditions, piperine forms a hydrogen bond with Ser-155, polar interactions with Ser-155, Thr-140, Ser-89 and Asp-97, and hydrophobic interactions with Tyr-88, Leu-85, Trp-84, Tyr-80, Ile-99, Met-100, Ile-153, Val-59, Ile-57, Val-75, Met-72, Trp-111, Phe-126, Met-135, Phe-115 and Ala-130 (Figure 9B). For a big grid box, piperine binds through polar interactions with Ser-89, Ser-155 and Asp-97, Pi-Pi stacking with Tyr-80, and hydrophobic interactions with Met-72, Val-75, Ala-94, Ile-99, Met-100, Trp-111, Tyr-80, Tyr-88, Leu-85, Trp-84, Phe-126, Ile-153, Val-59, Ile-57 (Figure 9D). Interestingly, the accommodation of piperine using a small or big grid box is very distinct, in a small grid as for C6-HSL the amide bond appears inside and at the bottom the pore of the active site, but in a big grid box the amide bond appears outside of the same pore (Figure 9B, D).

Applying the small grid box modelling, trichostachine was found to form a hydrogen bond with Ser-155, polar interactions with Ser-89, Asp-97, Thr-140 and Ser-155, hydrophobic interactions with Tyr-88, Leu-85, Trp-84, Met-135, Ala-130, Phe-126, Phe-115, Trp-111, Ile-99, Met-100, Ile-153, Ile-57, Tyr-80, Val-59, Val-75 and Met-72 (Figure 9C). Using a big grid box, trichostachine forms a hydrogen bond with Tyr-80, polar interactions with Ser-89, Ser-155 and Aps-97, hydrophobic interactions with Met-72, Val-75, Tyr-88, Leu-85, Trp-84, Tyr-80, Trp-111, Phe-126, Met-100, Ile-99, Ala-94, Val-59 and Ile-153 (Figure 9E). As well as for piperine, the accommodation of trichostachine in a small or in a big box results in a 180° rotation of the molecule inside the pore of the active site (Figure 9C, E).

It can be noticed that C6-HSL and the two piperamides, under small grid box parameters, commonly binds to CviR trough Tyr-80, Trp-84, Asp-97, Ser-155, Ile-57, Val-59, Met-100, Ile-99, Trp-111, Phe-126, Phe-115, Met-135, Ala-130, Leu-85, Tyr-88, Met-72, Val-75 and Ile-153 (Figure 9). As is described here, in previous docking studies it has been reported that C6-HLS binds to CviR trough hydrogen bonds and polar interactions with Asp-97, Trp-84, Ser-155, and trough hydrophobic contacts with Trp-111, Phe-126 and Tyr-80 (Sun et al., 2016; Ravichandran et al., 2018). Under the same conditions, piperine and trichostachine present exclusive interactions with Thr-140 and Ser-89.

For big grid box conditions, C6-HSL and the two piperamides commonly binds to CviR trough Tyr-80, Trp-84, Ser-155, Val-59, Met-100, Ile-99, Trp-111, Phe-126, Leu-85, Tyr-88, Met-72, Val-75 and Ile-153. Piperine and trichostachine interacts exclusively with Ser-89 and Ala-94. C6-HSL and piperine present an exclusive bond trough Ile-57 (Figure 9). By comparing the molecular docking results for QS modulatory compounds here described and the previous ones (Sun et al., 2016; Ravichandran et al., 2018; Chang et al., 2019; Rivera et al., 2019), it is possible to identify some key CviR residues involved in QS modulatory compounds perception: Asp-97, Ser-155, Tyr-80, Trp-84, Tyr-88, Phe-115, Phe-126, Val-75, Met-135 and Leu-85. Therefore, future search for CviR QS active compounds could be focused on those that interact with these residues.

## 4 Conclusions

It was demonstrated that piperine and trichostachine are the main components responsible for the QQ activity of the *P. nigrum* extract against *C. violaceum* CV026. The effective QQ concentrations were 0.5 g/L, 30 mg/L and 50 mg/L for *P. nigrum* extract, piperine and trichostachine, respectively. Molecular docking analysis shows that piperine and trichostachine are capable to accommodate in the active site of CviR with docking scores of -7.828 and -8.426, respectively. Further, it was observed that these compounds modulate the QS of *C. violaceum* CV026, but they did not affect the QS of *P. aeruginosa* PAO1. Data here described exhibit the potential of piperamides as modulators of QS dependent on AHLs, not previously reported.

## Declarations

### Author contribution statement

J. Vázquez-Martínez, J. Molina-Torres: Conceived and designed the experiments; Analyzed and interpreted the data; Contributed reagents, materials, analysis tools or data; Wrote the paper.

G. V Buitimea-Cantúa: Performed the experiments; Analyzed and interpreted the data; Contributed reagents, materials, analysis tools or data.

J. M Gutierrez-Villagomez, E. Ramírez-Chávez, J. P García-González: Analyzed and interpreted the data; Contributed reagents, materials, analysis tools or data.

### Funding statement

This work was supported by SEP-TecNM (Mexico) grant number 5181.19P.

### Competing interest statement

The authors declare no conflict of interest.

### Additional information

No additional information is available for this paper.

## Appendix A Supplementary data

Vazquez-Martinez et al_Supplementary material Vazquez-Martinez et al_Supplementary material
