# Supplementary material for: Bioautography and GC-MS based identification of piperine and trichostachine as the active quorum quenching compounds in black pepper
Source: Heliyon. 2020 Jan 2;6(1):e03137. doi: 10.1016/j.heliyon.2019.e03137 (PMC6948270; doi:10.1016/j.heliyon.2019.e03137)
Supplement: Vazquez-Martinez et al_Supplementary material [file mmc1.pdf]

Supplementary Data

Bioautography and GC-MS based identification of piperine and trichostachine as the active quorum quenching compounds in black pepper

Juan Vázquez-Martínez, Génesis V. Buitemea-Cantúa, Juan Manuel Gutierrez-Villagomez, Julia P. García-González, Enrique Ramírez-Chávez, Jorge Molina-Torres

1.1. Effect of *Piper nigrum* extract, piperine and trichostachine on growth and violacein production by *Chromobacterium violaceum* CV026

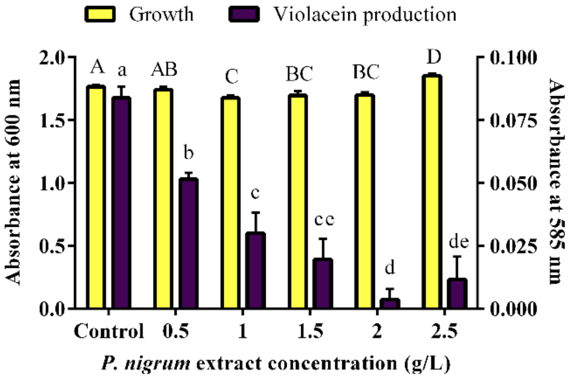

Supplementary Figure 1. Effect of *P. nigrum* extract on growth and violacein production by *C. violaceum* CV026. Data are shown as cell density (yellow bars) and violacein (violet bars) absorbance at 600 and 585 nm, respectively. Bars correspond to the mean values of five replicates and the error bars correspond to the standard deviation of the mean. Different capital letters indicate significant differences ($p < 0.05$) for growth data, and different lowercase letters indicate significant differences ($p < 0.05$) for violacein production data. Means with at least one common letter are not significantly different.

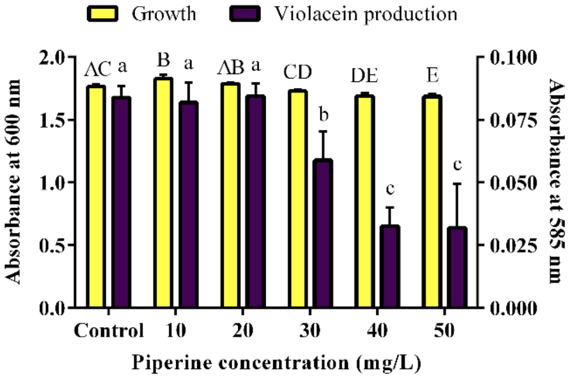

Supplementary Figure 2. Effect of piperine on growth and violacein production by *C. violaceum* CV026. Data are shown as cell density (yellow bars) and violacein (violet bars) absorbance at 600 and 585 nm, respectively. Bars correspond to the mean values of five replicates and the error bars correspond to the standard deviation of the mean. Different capital letters indicate significant differences ($p < 0.05$) for growth data, and different lowercase letters indicate significant differences ($p < 0.05$) for violacein production data. Means with at least one common letter are not significantly different.

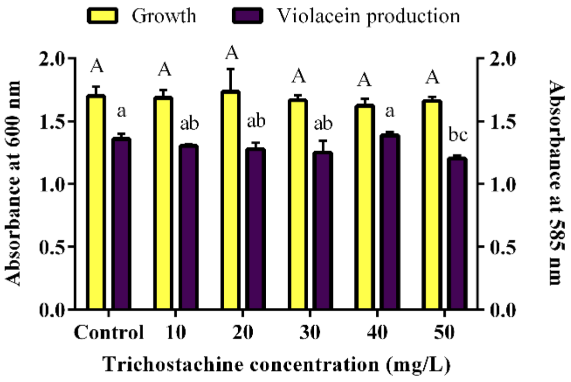

Supplementary Figure 3. Effect of trichostachine on growth and violacein production by *C. violaceum* CV026. Data are shown as cell density (yellow bars) and violacein (violet bars) absorbance at 600 and 585 nm, respectively. Bars correspond to the mean values of five replicates and the error bars correspond to the standard deviation of the mean. Different capital letters indicate significant differences ($p < 0.05$) for growth data, and different lowercase letters indicate significant differences ($p < 0.05$) for violacein production data. Means with at least one common letter are not significantly different.

1.2. Effect of *Piper nigrum* extract, piperine and trichostachine on biofilm production by *Pseudomonas aeruginosa* PAO1

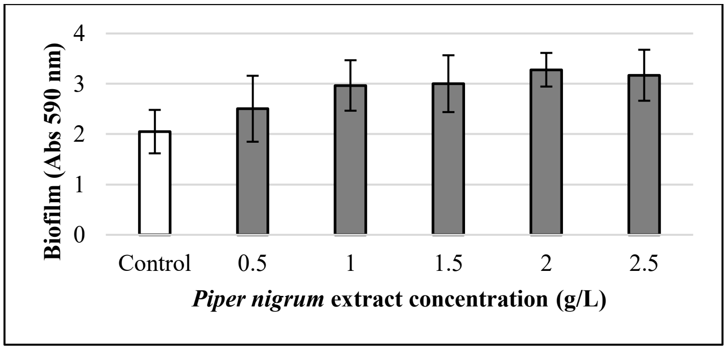

Supplementary Figure 4. Effect of *Piper nigrum* extract on biofilm production by *Pseudomonas aeruginosa* PAO1. Biofilm production was estimated as absorbance at 590 nm of crystal violet solution after extraction from stained biofilms obtained from each treatment. No significative differences among treatments were obtained ($p = 0.0894$). Solid bars indicate the mean of 5 replicates, error bars indicate the standard deviation of the mean.

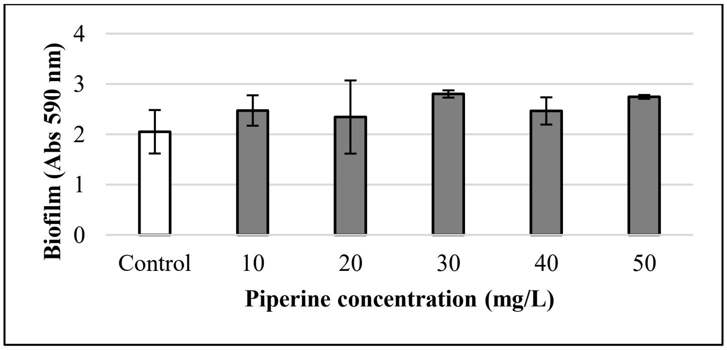

Supplementary Figure 5. Effect of piperine on biofilm production by *P. aeruginosa* PAO1. Biofilm production was estimated as absorbance at 590 nm of crystal violet solution after extraction from stained biofilms obtained from each

treatment. No significative differences among treatments were obtained (p = 0.2530). Solid bars indicate the mean of 5 replicates, error bars indicate the standard deviation of the mean.

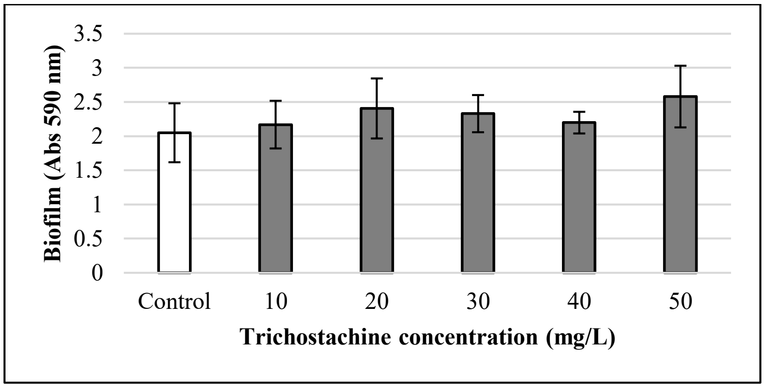

Supplementary Figure 6. Effect of trichostachine on biofilm production by *P. aeruginosa* PAO1. Biofilm production was estimated as absorbance at 590 nm of crystal violet solution after extraction from stained biofilms obtained from each treatment. No significative differences among treatments were obtained (p = 0.5673). Solid bars indicate the mean of 5 replicates, error bars indicate the standard deviation of the mean.

1.3. Effect of *Piper nigrum* extract, piperine and trichostachine on pyocyanin production by *Pseudomonas aeruginosa* PAO1

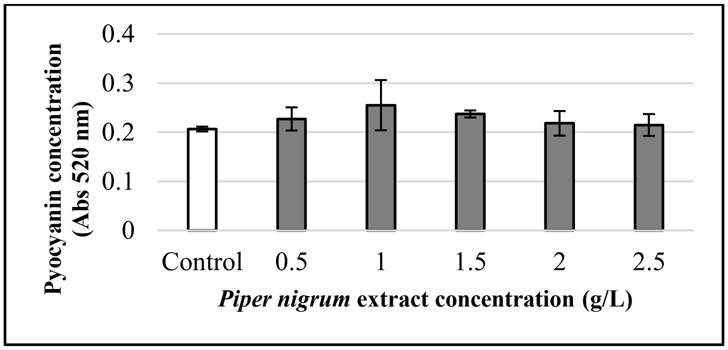

Supplementary Figure 7. Effect of *Piper nigrum* extract on pyocyanin production by *P. aeruginosa* PAO1. Pyocyanin production was estimated as absorbance at 520 nm of extracted pyocyanin from cultures of each treatment. No significative differences among treatments were obtained (p = 0.3375). Solid bars indicate the mean of 5 replicates, error bars indicate the standard deviation of the mean.

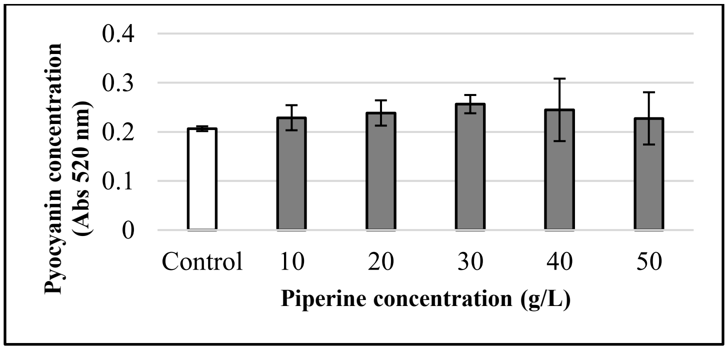

Supplementary Figure 8. Effect of piperine on pyocyanin production by *P. aeruginosa* PAO1. Pyocyanin production was estimated as absorbance at 520 nm of extracted pyocyanin from cultures of each treatment. No significative differences among treatments were obtained (p = 0.689). Solid bars indicate the mean of 5 replicates, error bars indicate the standard deviation of the mean.

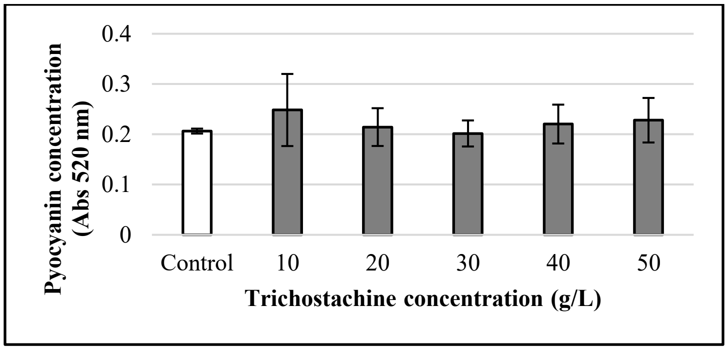

Supplementary Figure 9. Effect of trichostachine on pyocyanin production by *P. aeruginosa* PAO1. Pyocyanin production was estimated as absorbance at 520 nm of extracted pyocyanin from cultures of each treatment. No significative differences among treatments were obtained ($p = 0.7863$). Solid bars indicate the mean of 5 replicates, error bars indicate the standard deviation of the mean.

1.4. Comparison between GC-MS data of the identified piperine and the piperine standard

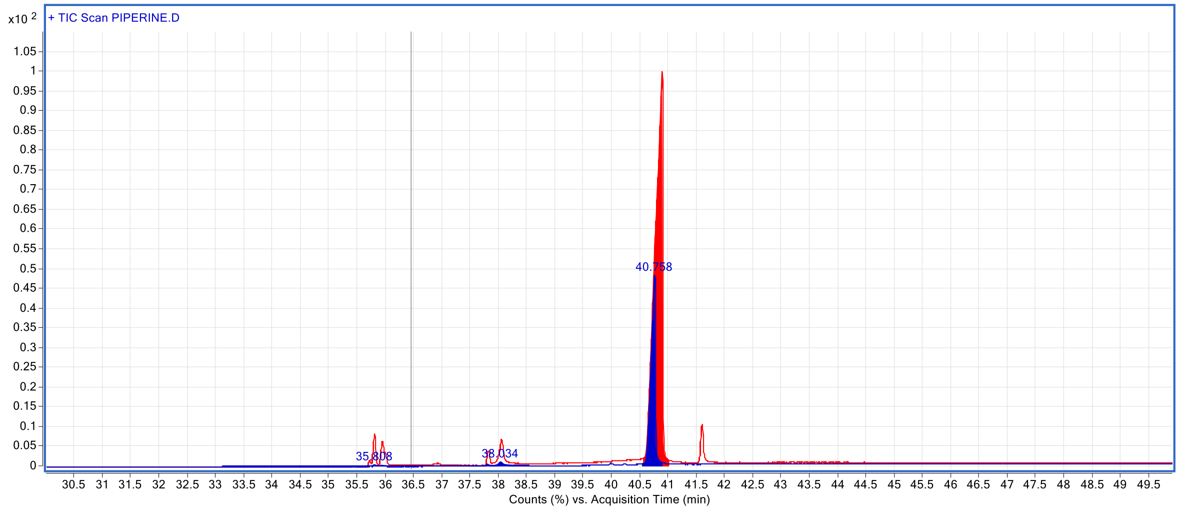

Supplementary Figure 10. Comparison between the purified piperine from the TLC band at Rf = 0.21 chromatogram (red) and the 97% purity piperine standard chromatogram (blue).

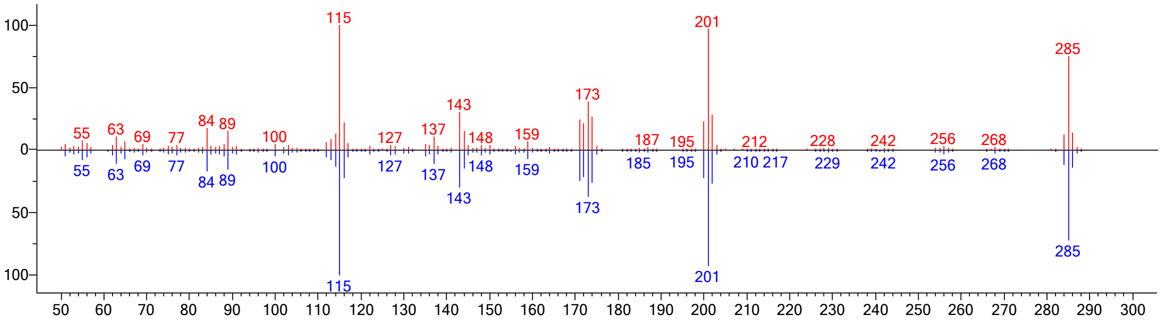

Supplementary Figure 11. Comparison between the purified piperine from the TLC band at Rf = 0.21 mass spectrum (red) and the 97% purity piperine standard mass spectrum (blue).
